# Changes in Salivary Parameters of Oral Immunity after Biologic Therapy for Inflammatory Bowel Disease

**DOI:** 10.3390/life11121409

**Published:** 2021-12-16

**Authors:** Kacper Nijakowski, Rafał Rutkowski, Piotr Eder, Katarzyna Korybalska, Janusz Witowski, Anna Surdacka

**Affiliations:** 1 Department of Conservative Dentistry and Endodontics, Poznan University of Medical Sciences, 60-812 Poznan, Poland; annasurd@ump.edu.pl; 2 Department of Pathophysiology, Poznan University of Medical Sciences, 60-806 Poznan, Poland; rrutkowski@ump.edu.pl (R.R.); koryb@ump.edu.pl (K.K.); jwitow@ump.edu.pl (J.W.); 3 Department of Gastroenterology, Dietetics and Internal Diseases, Poznan University of Medical Sciences, 60-355 Poznan, Poland; piotreder@ump.edu.pl

**Keywords:** inflammatory bowel disease, biologic therapy, saliva, biomarkers, myeloperoxidase, immunoglobulin A, oral immunity, oral health, Crohn’s disease, ulcerative colitis

## Abstract

We previously observed that inflammatory bowel disease (IBD) may compromise oral host defense, as assessed by decreased salivary levels of immunoglobulin A (IgA) and myeloperoxidase (MPO). Biologic therapy with inhibitors of cytokines or adhesion molecules is increasingly used for patients with IBD. Little is known, however, about how this treatment modality affects the release and properties of saliva. Here, we aimed to determine how biologic therapy in patients who had not responded to previous standard treatment with conventional drugs affected the salivary concentration of IgA and MPO. To this end, unstimulated whole mixed saliva was collected before treatment or after 10–12 weeks of therapy from 27 patients with Crohn’s disease (CD) and 24 patients with ulcerative colitis (UC). After the induction phase of therapy with biologics, salivary levels of IgA and MPO increased significantly in UC, but not in CD patients. These increases were approximately 8-fold and 6-fold, for IgA and MPO, respectively. Moreover, these effects occurred in UC patients who responded successfully to therapy, but not in those who failed to improve. Furthermore, the relative increases in salivary IgA and MPO correlated with the relative decrease in UC severity, as assessed by the Mayo scale. These data indicate that the successful therapy with biologics in UC patients results also in improved oral host defense. However, it remains to be determined why such an effect does not occur during therapy for CD.

## 1. Introduction

Despite many studies and research efforts over the years, the actual etiopathogenesis of inflammatory bowel disease (IBD) is still unknown. The two major forms of IBD are Crohn’s disease (CD) and ulcerative colitis (UC). According to the Guidelines of the Working Group of the National Consultant in Gastroenterology and the Polish Society of Gastroenterology for the management of patients with Crohn’s disease and ulcerative colitis, as well as modified recommendations of the European Crohn’s and Colitis Organisation (ECCO), the primary goal of treatment for IBD should be to achieve and then maintain clinical remission without the use of steroids. The first-line treatment for moderate IBD is based primarily on 5-aminosalicylic acid (5-ASA) derivatives (mesalazine and sulfasalazine) with or without corticosteroids. In cases of 5-ASA treatment failure and steroid resistance, steroid dependence or intolerance, thiopurines can be used [1,2,3,4,5,6].

For patients refractory to conventional therapy, the use of tumor necrosis factor-alpha (TNFα) inhibitors (infliximab, adalimumab, and certolizumab pegol) is recommended. Other biologic therapies may include vedolizumab (a monoclonal antibody against α4β7 integrin) and ustekinumab (a monoclonal antibody against the p40 subunit of cytokines IL-12 and IL-23) [7]. All biologics are administered by the intravenous route, except for adalimumab administered by the subcutaneous route and subsequent subcutaneous doses of ustekinumab (after the first intravenous dose). At the time of the study, the treatment of patients with Crohn’s disease with infliximab and adalimumab and with ulcerative colitis with infliximab and vedolizumab was reimbursed in Poland.

In view of the current pandemic situation, a special Position Paper of the Polish Society of Gastroenterology and the National Consultant in Gastroenterology on the management of patients with inflammatory bowel disease in the era of the COVID-19 pandemic was published last April [8]. It is recommended to continue the current treatment in patients in clinical remission. The predominance of subcutaneously administered biologic drugs has been suggested due to the shorter length of stay in the facility and the possibility of administration at home, so this should be considered when implementing a new therapy. An additional factor indicating adalimumab as the anti-TNF-α drug of first choice is the lower risk of antibody formation compared with infliximab. However, it is not recommended to switch from the current intravenous formulation to another subcutaneous formulation because of the increased risk of disease exacerbation. In contrast, vedolizumab is preferred for the treatment of older patients because it is less likely to increase the risk of concomitant infections. In addition, high doses of steroids should be avoided with the consideration of reducing the current dose. However, there is no clear evidence of an increased risk of SARS-CoV-2 infection when patients routinely take the drugs.

The effect of IBD on salivation and the properties of saliva is only partially under-stood. The salivary concentrations of a number of different molecules have been shown to change during IBD [9]. These include primarily mediators of inflammation and oxidative stress. Interestingly, we recently observed that in patients with long-lasting IBD unresponsive to conventional treatment, the concentration of some effectors of immunity was significantly lower than in healthy controls [10]. This effect was rather surprising, given that other studies [11,12,13,14] reported mainly on increased levels of several salivary components in IBD. We hypothesized that such a direction of changes could be related to immunosuppression caused by previous treatment attempts, rather than reflect the degree of inflammation induced by the disease.

Of the many components of saliva, immunoglobulin A (IgA) and myeloperoxidase (MPO) exemplify the mediators of local immunity and host defense. IgA has a defensive role by the opsonization and agglutination of bacteria, and preventing their adhesion to the epithelium of the gastrointestinal tract mucosa, including the oral cavity [15,16]. Altered IgA levels may predispose bacterial invasion into the mucosa, responsible for the development of erosions and ulcers [17]. Myeloperoxidase plays a role in microbial killing by neutrophils, but may also contribute to inflammation-associated tissue damage [18,19]. It is suggested that MPO concentrations can detect the healing of intestinal mucosa [20]. We therefore chose IgA and MPO as the parameters of oral immunity to monitor the biologic treatment in IBD.

Our study aimed to determine how biologic drugs used in induction therapy would affect the salivary biochemical parameters and how these would be related to the clinical status in IBD patients.

## 2. Materials and Methods

### 2.1. Study Participants

The study group included adult patients of both sexes, with inflammatory bowel diseases, qualified for biological treatment in the Department of Gastroenterology, Dietetics and Internal Medicine, Poznan University of Medical Sciences, between January 2019 and March 2020. All participants must have given their informed consent. Further patient recruitment was prevented by the outbreak of the COVID-19 pandemic. Of 51 patients with IBD analyzed, 27 patients had CD and 24 patients had UC. The diagnosis was based on clinical symptoms and endoscopic examination according to standard criteria [3,5]. Patients with concomitant autoimmune diseases (including diabetes [21]) were not included.

Patients with active, moderate to severe disease, not responding to previous conventional full-dose therapy or showing intolerance to such therapy (e.g., allergic reactions) were qualified for biological treatment. At the time of inclusion in the biologic treatment program, each patient was treated with fixed maximum doses of tolerated drugs. Patients (*n* = 20) who had previously been treated with biologics were included in the analysis if the time elapsed from previous therapy was >18 months [4,5].

The patients were examined before and after the induction phase of therapy, i.e., after 10–14 weeks. The Crohn’s Disease Activity Index (CDAI) scale [22] and the modified Mayo scale [23] were used to estimate the clinical activity of Crohn’s disease and ulcerative colitis, respectively. Clinical remission in individual scales (below 150 points for CD and below 2 points for UC) or a decrease in disease activity by a minimum of 100 points in CD patients or 3 points in UC patients was assumed to be the condition for determining a positive response to treatment [24,25].

At both time points of the study, routine laboratory tests were performed. Fasting venous blood samples were collected in the morning. On the same day, the following laboratory parameters were determined: C-reactive protein, white blood cells, neutrophils, red blood cells, hemoglobin, hematocrit, mean corpuscular volume and platelets.

### 2.2. Saliva Collection and Analysis

Oral health was examined in all individuals using routine methods by the same investigator (K.N.). Oral hygiene (approximal plaque index, API [26]; plaque index, PlI [27]) and periodontal tissues (gingival index, GI [28]; sulcus bleeding index, SBI [29]; probing periodontal depth, PPD) were assessed. Patients with periodontal disease or other overt inflammatory lesions in the oral cavity, and patients taking medications known to affect salivation [30] were not included.

Unstimulated whole mixed saliva was collected before and after the induction phase of therapy, as previously described [31]. Briefly, saliva was collected in the morning at least 2 h after a meal by passive drooling over 20 min. The saliva collected was immediately analyzed for pH and volume, then centrifuged to remove any debris, aliquoted and placed at −80 °C until assayed.

Salivary concentrations of myeloperoxidase and IgA were measured with enzyme-linked immunosorbent assays (respectively, DY3174, Bio-Techne, R&D Systems, Minneapolis, MN, USA; and DEXK276, Demeditec Diagnostics, Kiel, Germany), as per manufacturer instructions. Total protein salivary concentration was measured with the Bradford method using Bio-Rad Protein Assay Dye Reagent (Bio-Rad, Munich, Germany) [32].

### 2.3. The Statistical Analysis

Since the data did not follow the normal distribution (as determined by the Shapiro–Wilk test), medians and quartile ranges were used for descriptive statistics, and the non-parametric the Mann–Whitney test or the Wilcoxon test were used for comparisons. For qualitative variables a two-sided Fisher exact test was performed.

Changes in parameters of disease activity and selected salivary biomarkers were calculated by subtracting the initial value from the final value (after the induction phase). The association between them was evaluated with the Spearman rank correlation.

The significance level for all analyses was set at 0.05. The statistical analysis was performed using Statistica 13.3 (Statsoft, Cracow, Poland).

## 3. Results

### 3.1. Patients Characteristics

Table 1 shows the basic characteristics of the study group, including the clinical response to the induction therapy with the implemented biologic drug. The largest number of patients, regardless of the IBD form, were qualified for treatment with infliximab (IFX). While using this drug, satisfactory clinical response was observed in ¾ of CD patients and in ⅔ of UC patients.

Table 2 presents the parameters of oral health in IBD patients. The study group represented good oral hygiene status and no significant differences were observed between the established subgroups. All patients manifested healthy periodontal status, with only occasional mild localized gingivitis. As expected, the periodontal status indices correlated strongly with the oral hygiene indices (not shown).

### 3.2. Comparison of IBD Activity before and after the Induction Phase of Biologic Therapy

As expected, the introduction of biologic therapy resulted in a significant clinical improvement in patients with both CD and UC, as reflected by appropriate clinical indices (Table 3). After the induction phase of therapy, the clinical activity of the disease had already decreased significantly. This effect was associated with decreases (although not always formally significant) in CRP levels, total leukocyte, neutrophil, and platelet numbers, as well as increases in red blood cell count, hematocrit and hemoglobin concentration.

### 3.3. Comparison of Salivary Properties in IBD Patients before and after the Induction Phase of Biologic Therapy

The induction of biologic therapy for both Crohn’s disease and ulcerative colitis led to an increase in both pH and the flow rate of unstimulated saliva (Table 4). It also resulted in increased salivary concentrations of IgA and MPO (Table 5). These, however, reached statistical significance only in the UC group.

### 3.4. Comparison of Salivary Properties before and after the Induction Phase of Biologic Therapy in IBD Patients with or without Successful Response to Treatment

To see whether the changes observed were related to the response to therapy, the patients were stratified according to the treatment outcome (Table 6 and Table 7). These comparisons showed that the increases in pH, salivary flow rate and IgA and MPO concentrations seen in UC patients were confined to patients with a positive response to therapy. In contrast, there were no difference between CD patients with a successful and unsuccessful response to therapy. Accordingly, only in patients with UC, the biologic therapy resulted in changes in salivary IgA and MPO levels that correlated inversely with the level of disease activity (Table 8). These patients showed also a significant positive correlation between changes in salivary MPO concentration and serum hemoglobin (R = 0.423, *p*-value = 0.040). There were, however, no other significant correlations with changes in salivary IgA and MPO (not shown).

## 4. Discussion

In our recent study [10], we observed that salivary levels of myeloperoxidase and to a lesser extent of IgA were decreased in UC patients compared to both healthy controls and patients with CD. We hypothesized that such a difference could be related to previous dual immunosuppression regimens that were more commonly used by UC patients. Here, we show that the induction phase of subsequent therapy with biologics resulted in a significant increase in salivary IgA and MPO in UC but not in CD patients. Importantly, this effect occurred only in UC patients who responded satisfactorily to therapy.

According to this concept, the therapy with biologics restored the oral host defense that was compromised by prior immune suppression. In this respect, it was observed that the use of glucocorticoids decreased neutrophil production, which was associated with decreased concentrations of myeloperoxidase in body fluids, including the sputum [33].

In contrast to our observations, several other studies reported increased IgA in patients with active IBD [11,34,35,36]. However, this effect could be attributed to the presence of abnormalities in oral mucosa, which were absent in our group of patients.

Interestingly, we observed an increase in the pH of saliva in UC patients who responded to therapy. It needs to be determined whether such an effect can be at least partly related to the increase in MPO observed in these patients. This is because MPO in neutrophils is known to create and maintain an alkaline milieu important for effective phagocytosis [37].

It is difficult to relate observations of the present study to earlier reports, as previous studies did not seem to take into account the history of immunosuppression typically used in IBD patients. Furthermore, in our study, we found no significant correlation between salivary MPO and IgA with disease activity assessed before the initiation of therapy. This may be because the indexes of disease severity were generally high at this point. In addition, the disease activity scales have strong subjective components, which may limit their relationship with biochemical parameters.

Fecal calprotectin is commonly used for monitoring the response to treatment and healing of the intestinal mucosa [38,39]. In the only previous study examining the effect of biologic therapy for IBD on salivary parameters, Majster et al. [40] compared calprotectin levels in saliva before and after therapy. They found no significant differences in calprotectin levels in both unstimulated and stimulated saliva following 10–12 weeks of therapy. They did, however, observe a profound decrease in serum calprotectin as a result of therapy. This discrepancy may indicate that the levels of calprotectin in saliva correspond only weakly to the serum calprotectin concentration.

In this respect, Majster et al. [41] compared the levels of inflammatory cytokines in the saliva and blood from IBD patients, and related them to disease activity. In contrast to significant changes in the serum levels of many cytokines, there were only slight changes in the saliva that did not associate with disease activity.

Our research model with therapeutic intervention (induction phase of biologic treatment) had a relatively homogeneous study group in terms of oral health status. Among the few studies performed to date on the release and properties of saliva in IBD, our investigation is unique in that it assessed the direct impact of biologic therapy in patients with both UC and CD. In this respect, the observed differences between these forms of IBD are puzzling. They can be partly related to the pathobiology of the disease itself but also to the immunomodulatory effect of therapy, as the greatest differences occurred in UC patients who successfully responded to treatment. The limitations of the study included the fact that further patient recruitment to increase the power of the study was hampered by the COVID-19 pandemic. For the same reason, the direct supervision of saliva sampling was not always possible. Therefore, it is desirable for further studies to confirm the relationships observed. Furthermore, it would be useful to correlate the levels of markers determined in saliva with their levels in blood serum.

## 5. Conclusions

The relationship between clinical response to biologic therapy and salivary parameters of oral immunity in patients with inflammatory bowel disease cannot be clearly established. As a result of induction therapy, only patients with ulcerative colitis showed a significant increase in salivary IgA and myeloperoxidase to levels comparable to the controls. In patients with Crohn’s disease, as well as in patients with no clinical response, there were no significant changes in the measured levels of salivary markers. Our investigation demonstrated the potential use of non-invasive diagnostics with saliva to monitor the course of biological treatment in IBD patients, although further studies are required.

## Figures and Tables

**Table 1:** Basic parameters describing the study group by disease form—Crohn’s disease (CD) and ulcerative colitis (UC).

Parameter	CD *n* = 27	UC *n* = 24	*p*-Value ^#^
Gender, female, *n* (%)	10 (37.0)	7 (29.2)	0.767
Age, years, M [Q1–Q3]	34.0 [28.0–48.0]	32.0 [24.0–40.5]	0.171
Smokers, *n* (%)	7 (25.9)	1 (4.2)	0.053
Previous combined immunosupression (steroids + thiopurines), *n* (%)	7 (25.9)	11 (45.8)	0.156
Disease duration, years, M [Q1–Q3]	8.5 [6.0–12.0]	5.0 [3.0–10.0]	0.125
IFX, *n* (%)	16 (59.3)	15 (62.5)	>0.999
Clinical response for IFX, *n* (%)	12 (75.0)	10 (66.7)	0.704
Other biologics *, *n* (%)	11 (40.7)	9 (37.5)	>0.999
Clinical response for other biologics *, *n* (%)	11 (100.0)	7 (77.8)	0.190

Legend: IFX, infliximab; *adalimumab (ADA) for CD and vedolizumab (VDZ) for UC; ^#^ the Mann–Whitney test for quantitative variables and the two-sided Fisher exact test for qualitative variables.

**Table 2:** Oral parameters describing the study group by disease form—Crohn’s disease (CD) and ulcerative colitis (UC): M [Q1–Q3].

Parameter	CD	UC	*p*-Value ^#^
Approximal plaque index (%)	23.7 [11.5–37.5]	36.8 [22.3–47.4]	0.145
Plaque index	0.3 [0.2–0.4]	0.4 [0.2–0.6]	0.189
Sulcus bleeding index (%)	0.0 [0.0–0.0]	0.0 [0.0–4.1]	0.242
Gingival index	0.3 [0.3–0.3]	0.3 [0.2–0.5]	0.054
Probing periodontal depth (mm)	1.3 [1.2–1.6]	1.4 [1.2–1.5]	0.624

Legend: ^#^ the Mann–Whitney test.

**Table 3:** Clinical and blood morphology parameters in patients with Crohn’s disease (CD) and ulcerative colitis (UC) before and after the induction phase.

Parameter		Before Induction Phase M [Q1–Q3]	After Induction Phase M [Q1–Q3]	*p*-Value
Disease severity (CDAI for CD and Mayo scale for UC)	CD	289.9 [203.0–332.4]	118.2 [60.0–203.5]	<0.001 *
	UC	9.0 [8.5–10.5]	4.0 [1.0–7.0]	<0.001 *
BMI (kg/m^2^)	CD	22.1 [19.6–24.5]	22.2 [20.2–25.4]	0.012 *
	UC	22.6 [19.7–25.7]	22.7 [19.9–26.5]	0.142
CRP (mg/L)	CD	13.3 [3.6–25.8]	6.1 [1.2–16.3]	0.144
	UC	5.1 [1.6–12.3]	2.8 [1.1–6.9]	0.391
WBC (×10^3^/µL)	CD	7.5 [6.0–11.7]	7.3 [5.3–8.8]	0.058
	UC	9.8 [7.2–10.7]	6.3 [5.6–7.8]	0.001 *
NEU (×10^3^/µL)	CD	5.1 [3.9–7.4]	4.0 [3.2–6.3]	0.170
	UC	7.6 [4.5–8.9]	3.5 [2.8–4.3]	0.004 *
RBC (×10^6^/µL)	CD	4.7 [3.9–4.9]	4.7 [4.4–5.0]	0.133
	UC	4.5 [3.5–5.0]	4.8 [4.2–5.2]	0.011 *
HGB (g/dL)	CD	12.2 [10.3–14.2]	13.7 [12.4–14.8]	0.003 *
	UC	13.0 [9.5–13.8]	13.5 [12.0–15.0]	0.003 *
HCT (%)	CD	38.0 [33.6–42.9]	41.2 [38.1–44.2]	0.009 *
	UC	39.4 [30.0–42.4]	40.1 [35.9–44.3]	0.016 *
MCV (fl)	CD	87.4 [76.4–89.9]	89.1 [85.5–91.0]	0.062
	UC	85.5 [84.1–89.1]	86.0 [84.0–88.8]	0.830
PLT (×10^3^/µL)	CD	340.0 [297.0–436.0]	300.0 [246.0–358.0]	0.006 *
	UC	378 [304.0–460.0]	335.0 [263.0–371.5]	0.209

* *p*-value < 0.05 for the Wilcoxon test. Legend: CDAI, Crohn’s Disease Activity Index; BMI, body mass index; CRP, C-reactive protein; WBC, white blood cells; NEU, neutrophils; RBC, red blood cells; HGB, hemoglobin; HCT, hematocrit; MCV, mean corpuscular volume; PLT, platelets.

**Table 4:** Basic features of salivation in patients with Crohn’s disease (CD) and ulcerative colitis (UC) before and after the induction phase.

Parameter	IBD Form	Before Induction Phase M [Q1–Q3]	After Induction Phase M [Q1–Q3]	*p*-Value
pH	CD	6.8 [6.5–7.0]	7.0 [6.7–7.1]	0.307
	UC	6.9 [6.5–7.0]	6.8 [6.7–7.2]	0.028 *
Flow rate (mL/min)	CD	0.4 [0.3–0.5]	0.5 [0.3–0.7]	0.091
	UC	0.3 [0.3–0.4]	0.3 [0.3–0.5]	0.236
Total protein (µg/mL)	CD	254.0 [131.6–559.0]	358.4 [132.5–507.5]	0.981
	UC	294.0 [181.6–406.4]	210.7 [59.0–383.7]	0.627

* *p*-value < 0.05 for the Wilcoxon test.

**Table 5:** Comparison of concentrations of selected salivary markers in patients with Crohn’s disease (CD) and ulcerative colitis (UC) before and after induction phase.

Parameter	IBD Form	Before Induction Phase M [Q1–Q3]	After Induction Phase M [Q1–Q3]	*p*-Value
IgA (µg/mL)	CD	52.1 [0.4–175.2]	112.3 [8.3–151.4]	0.381
	UC	10.9 [0.1–77.5]	80.1 [1.9–344.2]	0.021 *
Myeloperoxidase (ng/mL)	CD	57.2 [4.9–110.6]	78.1 [7.2–195.2]	0.773
	UC	9.0 [3.2–28.0]	55.4 [2.1–187.1]	0.030 *

* *p*-value < 0.05 for the Wilcoxon test.

**Table 6:** Basic features of salivation before and after the induction phase of biologic therapy in patients with Crohn’s disease (CD) and ulcerative colitis (UC) with or without clinical response to treatment.

Parameter	IBD Form	Clinical Response	Before Induction Phase M [Q1–Q3]	After Induction Phase M [Q1–Q3]	*p*-Value
pH	CD	+	6.9 [6.6–7.0]	6.9 [6.8–7.1]	0.446
		−	6.6 [6.5–6.9]	7.0 [6.7–7.0]	0.636
	UC	+	6.8 [6.6–6.9]	6.9 [6.7–7.3]	0.003 *
		−	6.9 [6.4–7.2]	6.8 [6.6–6.9]	0.463
Flow rate (mL/min)	CD	+	0.4 [0.4–0.5]	0.5 [0.3–0.7]	0.246
		−	0.4 [0.3–0.5]	0.3 [0.3–0.7]	0.314
	UC	+	0.3 [0.3–0.4]	0.4 [0.3–0.5]	0.026 *
		−	0.3 [0.3–0.8]	0.3 [0.2–0.3]	0.249
Total protein (µg/mL)	CD	+	337.7 [208.2–563.0]	364.2 [236.0–501.0]	0.711
		−	181.5 [34.7–199.9]	296.7 [91.0–611.5]	0.594
	UC	+	220.8 [140.0–336.0]	187.0 [36.0–377.0]	0.523
		−	817.0 [298.0–1050.0]	351.0 [81.9–922.0]	0.063

* *p*-value < 0.05 for the Wilcoxon test.

**Table 7:** Comparison of concentrations of selected salivary markers before and after the induction phase of biologic therapy in patients with Crohn’s disease (CD) and ulcerative colitis (UC) with or without clinical response to treatment.

Parameter	IBD Form	Clinical Response	Before Induction Phase M [Q1–Q3]	After Induction Phase M [Q1–Q3]	*p*-Value
Immunoglobulin A (µg/mL)	CD	+	59.3 [0.4–170.7]	110.9 [8.3–149.5]	0.831
		−	45.1 [6.2–175.2]	124.3 [46.7–151.4]	0.314
	UC	+	6.2 [0.2–46.4]	147.0 [33.4–367.8]	0.009 *
		−	59.8 [0.1–90.7]	3.0 [0.2–315.7]	0.917
Myeloperoxidase (ng/mL)	CD	+	69.0 [8.7–103.1]	74.2 [12.5–175.6]	0.948
		−	57.2 [1.8–115.3]	123.2 [3.9–305.5]	0.678
	UC	+	4.0 [3.1–16.5]	175.0 [11.8–199.7]	0.004 *
		−	23.9 [7.6–177.3]	8.1 [1.8–89.6]	0.499

* *p*-value < 0.05 for the Wilcoxon test.

**Table 8:** Spearman correlation coefficients (R) for changes in parameters of disease activity and assessed salivary biomarkers in patients with Crohn’s disease (CD) and ulcerative colitis (UC).

Parameter	∆ IgA (µg/mL)				∆ MPO (ng/mL)			
	CD		UC		CD		UC	
	R	*p*-Value	R	*p*-Value	R	*p*-Value	R	*p*-Value
∆ Disease severity (CDAI for CD and Mayo scale for UC)	−0.164	0.415	−0.421 *	0.041	−0.015	0.942	−0.546 *	0.006

* significant correlation for *p*-value < 0.05 according to the Spearman rank correlation. Legend: CDAI, Crohn’s Disease Activity Index; IgA, immunoglobulin A; MPO, myeloperoxidase; ∆, subtracting the initial value from the final value (after the induction phase).

## Data Availability

Data are available on request from the corresponding author.
